# Supplementary material for: GDNF receptor GFRα1 is necessary for the maintenance of dopaminergic neurons in the adult substantia nigra
Source: PLoS One. 2025 Sep 4;20(9):e0331369. doi: 10.1371/journal.pone.0331369 (PMC12410770; doi:10.1371/journal.pone.0331369)
Supplement: Attachment — Submitted filename: Response to Reviewers_Carrier-Ruiz.pdf [file pone.0331369.s002.pdf]

Response to Reviewers:

Dear Dr. Michael Francis Salvatore,

We thank you and the reviewers for their valuable comments. We have used your input to make the requested changes to the manuscript. Please find below the answers to each point raised by you and the reviewers. We believe that the changes made have improved the manuscript which we now hope will be acceptable for publication in PLOS ONE.

Sincerely,

Alvaro Carrier Ruiz, PhD

On behalf of all co-authors

Responses regarding Journal requeriments:

1. “Please ensure that your manuscript meets PLOS ONE's style requirements, including those for file naming.”

We have ensured that the manuscript meets PLOS ONE’s style requirements, with the most notable change being the placement of the Material and Methods section after the Introduction section.

2. “To comply with PLOS ONE submissions requirements, in your Methods section, please provide additional information regarding the experiments involving animals and ensure you have included details on (1) methods of sacrifice, and (2) efforts to alleviate suffering.”

As requested by PLOS ONE submition requirements, in our Methods section, we have included details regarding the methods of sacrifice, and efforts to alleviate suffering in the Mice subsection.

3. “We note that you have provided funding information that is not currently declared in your Funding Statement. However, funding information should not appear in the Acknowledgments section or other areas of your manuscript. We will only publish funding information present in the Funding Statement section of the online submission form. Please remove any funding-related text from the manuscript and let us know how you would like to update your Funding Statement.”

We have removed the funding information from the Acknowledgements Section of our manuscript. The information presented in the Funding Statement remains the same and correct.

4. “All PLOS journals now require all data underlying the findings described in their manuscript to be freely available to other researchers, either 1. In a public repository, 2. Within the manuscript itself, or 3. Uploaded as supplementary information.”

All data underlying the findings described in the manuscript have been uploaded as supplementary information.

5. "Please review your reference list to ensure that it is complete and correct."

We have added one new reference to the revised manuscript (number 33) and confirm that the list is complete and correct.

Response to Reviewer 1's points

Comments:

1. "In the methods section, please include a description for the mice which were used for the postnatal study (figure 1). The only mention of these mice seems to be in the tissue processing section (page 18; lines 297-299)."

As requested, we have included in the Mice subsection of the Materials and Methods section the description of the wild-type mice used for the postnatal examination of the SN and dSTR by *in situ* hybridization.

2. "It is noted that both males and females were used in this study. Were there any sex differences seen in the postnatal mRNA expression levels of Gfra1 and Ret in the mouse SN, postnatal Gfrα1 mRNA expression in the dorsal striatum, or the acute deletion of Gfra1 on adult SN dopaminergic neurons?"

We appreciate the reviewer's comment for bringing the possibility of sex differences to our attention, specially given that such differences do occur between men and women with Parkinson's Disease. In our dataset at least, we did not see any sex difference in the examined parameters. We have included in the Mice subsection of the Materials and Methods section a statement regarding this.

3. "The authors noted that tamoxifen injection in Gfrα1CreERT2/flox;Rosa26dTOM mice results in the loss of GFRα1 in all brain cells that normally express the receptor. Could the authors cite data highlighting loss of GFRa1 in key brain areas which may affect the survival of SN dopaminergic cells in a non-cell-autonomous fashion from their group or other groups which would help the reader to better put their data in context?"

We appreciate the reviewer's comment for bringing this up to our attention. As far as we are aware, there are no papers investigating the effect of GFRα1 signalling outside the STR-SN pathway affecting the survival of SN dopaminergic neurons. However, it is known that pedunculopontine nucleus excitatory cholinergic inputs might contribute to the survival of SN dopaminergic neurons in the adult brain. In addition, data from the Allen Brain Atlas shows that this area is positive for GFRα1 mRNA, but whether this

pathway is relevant to the excitatory cholinergic inputs remains to be addressed. We briefly comment this possibility at the end of the manuscript.

4. “The authors suggest that acute disruption of GFRα1 expression preferentially affects SN dopaminergic neurons projecting to the striatum. Based on work done by either this group or others, would (could) this loss result in behavioral effects (signs) mimicking Parkinson’s disease? Could the authors briefly speak to this theme as well?”

We appreciate the reviewer’s curiosity about the possibility of Parkinson’s Disease-like behavioral effects after the loss of GFRα1-expressing neurons from the SN. We have not done any nigro-striatal behavioral testing with these mice yet. Parkinson's disease becomes symptomatic only after more than 50% of striatal dopamine levels and 30–40% of SNpc neurons are lost. Since we have observed a reduction of 24% of the SN dopaminergic neurons projecting to the STR, these mice could be just under the threshold for manifestation of PD symptoms. This is something that will have to be investigated in future studies. We have discussed this possibility in the revised version of the Discussion.

Minor comments

1. “Correct the typo on Page 10 (Introduction [lines 50-51]): “The glycoprotein Syndecan-3 has also been proposed as a receptor fror GDNF family ligands (13),”.”

The typo has been corrected.

2. “Ensure the consistent use of abbreviations throughout the manuscript. For eg, dorsal striatum was defined as “dSTR” on page 11 (line 86). However, the term was repeated multiple times throughout the manuscript with and without the abbreviation.”

The inconsistent use of abbreviations has been corrected.

Response to Reviewer 2’s points:

“The authors should carefully describe and discuss limitations of their study.”

We appreciate the reviewer’s suggestion to carefully describe and discuss the limitations of our study. We have done modifications throughout the revised version of the manuscript in order to address the more relevant questions and we would like to highlight the following points of discussion brought up by the reviewer.

1. “First, their results are obtained in the mouse model and its translation to human dopaminergic neurons is not clear. Indeed, there are multiple studies showing

excellent disease modifying effects, including the reduction of aSyn aggregation, of GDNF in the rodent PD models, and yet the results of clinical trials are inconclusive.”

We appreciate the reviewer’s concern regarding the discrepancies between the preclinical and clinical trials data of GDNF treatment for PD. The inconclusive results from the clinical trials can be roughly divided into two categories, logistics and biological. Logistically, we can highlight the variability in GDNF delivery systems, statistical considerations and issues with patient grading and inclusion criteria. Biologically, the aberrant sprouting at the site of GDNF administration and GDNF dosing, which usually exceeds endogenous GDNF levels, are the main points of concern. Further investigation into the expression and activation of GFRα1 in the human nigro-striatal pathway could hopefully provide solutions to these issues. We have added a full new paragraph in the revised version of the discussion commenting about this.

2. “Second, the authors should highlight potential technical issues. For example, they could not exclude the possibility of incomplete recombination: some dTOM positive cells may still express Gfra1, and some Gfra1-null cells may not be dTOM positive.”

We appreciate the reviewer’s concern regarding the possibility of incomplete recombination in the GFRα1 transgenic mice. Indeed, since the cre-dependent dTOM reporter is not directly under the GFRα1 promoter, but originates from a Rosa26 allele, we cannot exclude the existence of dTOM positive cells that may still express Gfra1 and Gfra1-null cells that may not be dTOM positive. However, the co-localization controls between dTOM and GFRα1 antibody signals performed when we generated this transgenic line and shown in our previous report (Suárez-Fernández et. al., 2021) do not demonstrate any signs of incomplete recombination, thus, we don’t think it to be an issue in this case.

3. “The authors followed the fate of dopaminergic neurons by TH immunostaining only at a very short timepoint (3 days) after tamoxifen administration. The Gfra1 ablation may thus be just causing a (potentially only transient) down-regulation of TH expression. Fluorogold tracing results suggest the degeneration of neurons, but statistical analysis of these data is not shown, and it is hard to imagine that neurons would degenerate in such a short time frame. Also, potentially more neurons would degenerate at longer timepoints. The study would be more convincing if the authors had examined Gfra1 positive nigral dopaminergic neurons and striatal dopaminergic innervation at multiple timepoints after Gfra1 ablation.”

We appreciate the reviewer’s concern regarding the fate of the targeted dopaminergic neuros. To clarify, we investigated the fate of the neurons 3 days after <u>the last tamoxifen administration</u>, namely 7 days after the first tamoxifen injection. Data from a reference cited in the manuscript show that during the wave of cell death that occurs in SN dopaminergic neurons around P14, 2 days was sufficient to observe a five-fold increase in the number of apoptotic cells in the SN (Burke, 2003). Regarding the statistical analysis of the number of

FluoroGold traced neurons, those data are shown in Fig. 4B. We agree with the reviewer on the possibility that more neurons might degenerate at longer timepoints than the one investigated here. This is something we plan to address in future work, together with the molecular mechanisms of this celular decline in the SN of GFRα1 KO animals.

4. “The authors should demonstrate or, if it was shown before, provide a clear comment and reference about potential “leakiness” of CreERT2, by including a mouse group injected with oil+10% ethanol vehicle control.”

We appreciate the reviewer’s concern regarding the potential “leakiness” of the CreERT2 system. We are aware that it is generally assumed that Cre is excluded from the nucleus (and thereby unable to recombine loxP sites) in the absence of tamoxifen, but there are examples of ubiquitous Cre, pancreas-specific Cre and melanocyte-specifc Cre that do recombine sequences without induction, as described by The Jackson Laboratories (https://www.jax.org/news-and-insights/jax-blog/2013/september/a-dozen-facts-you-didnt-know-about-cre-lox). These cases of “Cre leakiness” appear to be very strain specific. In our 10+ years of experience using the Gfra1^{CreERT2} mouse strain, Cre induction independently of tamoxifen has not been an issue. We have done the appropriated vehicle injected mice in our previous paper using the same mouse line (Suárez-Fernández et. al., 2021) and have not observed relevant levels of non-specific recombination.

5. “Bar graphs should show individual datapoints (see https://doi.org/10.1038/s41551-017-0079).”

We appreciate the reviewer’s comment for bringing this up to our attention. All bar graphs now also show individual data points.

6. “The quality of immunostaining and RNAscope images (at least in the reviewers’ PDF) is very poor, especially red channels on Figs. 1 and 2.”

We appreciate the reviewer’s comment for bringing this up to our attention. We believe this is an issue with the automated PDF generated for the reviewers at the time of submission. The images we submited to the system have a very strong and clear red signal. We will pay extra attention to the images’s quality in the final version of the manuscript to avoid this problem from happening again.

7. “Line 95: “substantial” -> substantia”

The typo has been corrected

8. “Line 140: “septus” -> septum”

The typo has been corrected

Response to Reviewer 3's points:

1. "It is mentioned that "insertion of CreERT2 in the Gfra1 locus inactivates the targeted allele," and while there are several references cited, there wasn't any data presented demonstrating that this does in fact reduce expression of Gfra1. Furthermore, it would be worthwhile to demonstrate in the Gfrα1CreERT2/flox that Gfrα1 expression is truly ablated."

We appreciate the reviewer's concern regarding the effectiveness of the Gfrα1 ablation. We do not show those data here because they were already published in a previous paper from our group (Suárez-Fernández et. al., 2021). In that paper, we showed that mRNA and protein levels of GFRα1 decrease by approximately 50-60% in the *Gfrα1*$^{CreERT2/+}$ heterozygous mice and by 80-90% in the *Gfrα1*$^{CreERT2/flox}$ mice, so we didn't find it relevant to submit our own replicated data in this manuscript.

2. "One question I had after reading the manuscript is whether ablation of Gfrα1 expression actually leads to loss of neurons or just alters their programing such that they no longer express dopaminergic markers. I think it would be worth using a nondopaminergic marker or simply using DAPI staining to label the nuclei so you can determine if Gfrα1 ablation actually leads to loss neurons or not."

We appreciate the reviewer's suggestion about using a non-dopaminergic marker or simply DAPI to investigate the loss of neurons. However, in this study we observed evidence of the loss of these neurons when we quantified the number of FluoroGold positive cells in the SN (Fig. 4B). Since we perform the tamoxifen-dependent ablation of GFRα1 <u>one week after</u> the intrastriatal injection of FluoroGold, the SN dopaminergic neurons would already have been traced at the time of induction. Being that the case, the simplest explanation for the decrease of FluoroGold positive cells is that the neurons are being lost altogether rather than transiently reprogramed. Something that we plan to address in future work is the timeline and molecular mechanisms of this celular decline in the SN of GFRα1 KO animals.
