# Supplementary material for: GDNF receptor GFRα1 is necessary for the maintenance of dopaminergic neurons in the adult substantia nigra
Source: PLoS One. 2025 Sep 4;20(9):e0331369. doi: 10.1371/journal.pone.0331369 (PMC12410770; doi:10.1371/journal.pone.0331369)
Supplement: Attachment — Submitted filename: Response to Reviewers_Carrier-Ruiz_r2.pdf [file pone.0331369.s003.pdf]

Response to Reviewers:

Dear Dr. Michael Francis Salvatore,

We thank you and the reviewers for their valuable comments. We are pleased to have satisfactorily addressed all the reviewers' comments, resulting in an improved manuscript. We have used your new input to make the requested changes to the manuscript. Please find below the response to your raised point. We believe that the changes made have refined the manuscript, which we now hope will be acceptable for publication in PLOS ONE.

Sincerely,

Alvaro Carrier Ruiz, PhD

On behalf of all co-authors

Responses regarding Journal requeriments:

1. "Recommendation to cite specific previously published work."

We have reviewed and evaluated the recommended work cited by the editor and have found it to be relevant to be cited in our manuscript.

2. "Please review your reference list to ensure that it is complete and correct."

We have added one new reference to the revised manuscript (number 34) and confirm that the list is complete and correct.

Response to Editor's point

Comments:

1. "The authors rightly connect their work to Parkinson's disease, given what has been revealed with preclinical and clinical GDNF studies. GFR-α1 function in dopamine neurons has been far less studied, but arguably just as important as its cognate ligand. Again, I refer the authors to consider comments from reviewer 1 in points 3 and 4 from the first submission regarding GFR-α1 expression in the nigrostriatal pathway and potential application to Parkinson's. Kasanga et al (Exp Neurol, 2023) reported progressive GFRα1 loss that was in association with loss of nigrostriatal neurons. This loss was specific to nigrostriatal neurons. Moreover, GFRα1 was upregulated in astrocytes as the lesion progression continued. This finding seems to be very applicable to this study and may be useful in the discussion."

We appreciate the comment and thank the editor for bringing the mentioned report to our attention. The suggested study by Kasanga et al. (Exp Neurol, 2023) provides particularly valuable insight into the potential role of GFRα1 in

Parkinson's disease–related neurodegeneration. Using a PD model, their finding underscores a possible mechanistic link between receptor availability and neuronal vulnerability, which we investigated using our genetic GFRα1 ablation model. Furthermore, the reported upregulation of GFRα1 in astrocytes during lesion progression suggests an adaptive or compensatory glial response, raising new questions about neuron–glia interactions in disease progression and therapeutic strategies. Taking these findings into account and the previously addressed points of Reviewer 1, we have rewritten the last paragraph of the discussion to strengthen the translational relevance of our work to Parkinson's disease.
